# Liver Transplantation for Glycogen Storage Disease Type IV

**DOI:** 10.3389/fped.2021.633822

**Published:** 2021-02-19

**Authors:** Min Liu, Li-Ying Sun

**Affiliations:** ^1^Department of Liver Transplantation Center, Beijing Friendship Hospital, Capital Medical University, Beijing, China; ^2^National Clinical Research Centre for Digestive Diseases, Beijing Friendship Hospital, Capital Medical University, Beijing, China; ^3^Department of Intensive Care Unit, Beijing Friendship Hospital, Capital Medical University, Beijing, China

**Keywords:** glycogen storage disease type IV, glycogen branching enzyme, Andersen disease, liver transplantation, metabolism

## Abstract

Glycogen storage disease type IV (GSD IV) is a rare autosomal recessive disorder caused by glycogen–branching enzyme (GBE) deficiency, leading to accumulation of amylopectin–like glycogen that may damage affected tissues. The clinical manifestations of GSD IV are heterogeneous; one of which is the classic manifestation of progressive hepatic fibrosis. There is no specific treatment available for GSD IV. Currently, liver transplantation is an option. It is crucial to evaluate long–term outcomes of liver transplantation. We reviewed the published literature for GSD IV patients undergoing liver transplantation. To date, some successful liver transplantations have increased the quantity and quality of life in patients. Although the extrahepatic manifestations of GSD IV may still progress after transplantation, especially cardiomyopathy. Patients with cardiac involvement are candidates for cardiac transplantation. Liver transplantation remains the only effective therapeutic option for treatment of GSD IV. However, liver transplantation may not alter the extrahepatic progression of GSD IV. Patients should be carefully assessed before liver transplantation.

## Introduction

Glycogen storage disease type IV (GSD IV, Andersen disease, amylopectinosis, OMIM:232500) is an autosomal recessive disorder of glycogen metabolism (1). GSD IV is estimated to occur in 1 in 600 000 to 800 000 individuals worldwide (2). GSD IV is caused by deficiency of glycogen-branching enzyme (GBE), encoded by *GBE1* gene, leading to accumulation of amylopectin-like glycogen (polyglucosan) in affected tissues (3–6). More than 40 mutations in the *GBE1* gene have been found to cause GSD IV. *GBE1* is located on chromosome 3p14 and encodes a 702-amino-acid protein (7–9).

GSD IV is a rare form of glycogen storage disease that accounts for ~3% of glycogenosis (2). The accumulation of abnormal branched glycogen results in tissue damage. The clinical manifestations of GSD IV are heterogeneous with variable age of onset, severity and involvement of the liver, cardiac muscle neuromuscular system (3). The progressive hepatic type is the most common and classic form of GSD IV and is characterized by hepatomegaly that rapidly progresses to liver cirrhosis. Patients ultimately die from liver failure by 5 years of age, unless liver transplantation is performed (10, 11). Patients with a rare non-progressive hepatic form do not develop cirrhosis and survive to adulthood without liver transplantation (12–14). The neuromuscular form of GSD IV varies in onset (from fetal to adult age) and severity (4, 7, 15), which present with symptoms of myopathy, cardiomyopathy, and central and peripheral nervous system dysfunction (7, 16).The fatal infantile neuromuscular type is the most severe form of GSD IV (17, 18). Patients with the congenital neuromuscular form have severe muscle wasting, hypotonia, cardiomyopathy, respiratory distress, neuronal involvement and death in early infancy (19, 20). Neurological adult forms can present as isolated myopathy or as widespread upper and lower motor neuron lesions (adult polyglucosan body disease, APBD) (21). Liver biopsies have confirmed signs of cirrhosis and cells with periodic acid-Schiff (PAS) –stained, diastase-resistant inclusions, consistent with GSD IV. There is no specific treatment for GSD IV. Prognosis is unfavorable for patients with severe cardiomyopathy, associated neurological dysfunction and classic forms who do not undergo liver transplantation.

As there is no treatment to compensate for the deficient enzyme activity, additionally, the classic form of GSD IV usually leads to liver failure, and liver transplantation is the only effective treatment presently available for GSD IV with progressive liver disease (22). Since the first successful liver transplant for GSD IV in September 1984 (23), it has been increasingly used to treat GSD IV cases with liver failure and has improved survival. It is important to know the long–term outcomes of this therapeutic option. In this article, we review all GSD IV patients who underwent liver transplantation to explore the effectiveness and outcomes of liver transplantation for GSD IV.

## Methodology

The English–language literature about GSD IV patients undergoing liver transplantation was systematically reviewed in PubMed and in the references of relevant publications. The key words used included glycogen storage disease type IV, Andersen disease, amylopectinosis, type IV glycogenosis, glycogen-branching enzyme deficiency, and liver transplantation. Indications for liver transplantation, age at transplantation, follow-up period and complications were reviewed. Liver function post–transplantation, extrahepatic manifestations after transplantations, and clinical outcomes were analyzed to better clarify the clinical value of liver transplantation for GSD IV.

## Results

We summarized the characteristics and results of 24 patients with GSD IV who underwent liver transplantation between 1984 and 2017 (Table 1). There were 19 male patients with two sets of brothers (cases 7 and12, 8 and 11) and five female patients. The youngest was transplanted at 10 months of age and the oldest at 23 years. Two patients required a second liver transplantation: one for nonfunction of the graft 2 d after the first liver transplant, and one for antibody–mediated rejection due to ABO–incompatible live donor graft 6.2 mo after the first transplant. A living related transplantation was performed in seven patients. The indication for liver transplantation was based on the occurrence of progressive liver cirrhosis with evidence of portal hypertension in most cases; for one patient, liver cirrhosis was associated with liver adenoma, and for another, it was heart failure.

**Table 1:** Patients with GSD IV who underwent liver transplantation.

**References**	**Case**	**Sex**	**Age at LT (yr.)**	**Indications for LT**	**Complications**	**Follow-up/survival (yr.)**	**Liver function post-LT**	**Cardio-/myo-/neuropathy**
Aksu et al. (3)	1	F	23	HF LC	None	0.5	NA	cardiomyopathy
Choi et al. (24)	2	M	0.8	LF	None	1	normal	None
Willot et al. (25)	3	M	0.9	LF	Death^a^	6.3	abnormal	cardiomyopathy
	4	M	0.9	LF	Death^b^	1	normal	Cardiomyopathy myopathy
Sokal et al. (26)	5	M	1.2	LF	Death^b^	0.9	normal	Cardiomyopathy
Ban et al. (27)	6	F	2.3	LF	None	0.5	normal	NA
Selby et al. (23, 28)	7	M	2.6	LF	NA	13.2	normal	None
	8	M	0.9	LF	Death^c^	[7 days]		
	9	M	3.0	LF	Death^d^	0.1		
	10	M	3.8	LT	NA	11.9	normal	None
	11	M	1.8	LF	Death^e^	5.4	normal	
	12	M	1.7	LF	NA	8.8	normal	None
	13	M	2.8	LF	NA	8.7	normal	None
Dhawan et al. (29)	14	M	12	LF	Non-function of graft			None
				Re-transplant (2 days after 1st LT)^f^	NA	3.9		
Alshak et al. (30)Rosenthal et al. (31)	15	M	0.9	LF HA	Rejection Death^b^	2.5	normal	cardiomyopathy
Bruno et al. (7)	16	M	3	LF	mild hypotonia Arthrogryposis	3	normal	myopathy
Magoulas et al. (2)	17	F	0.8	LF	Sepsis Death^g^	0.3	normal	cardiomyopathy
Morioka et al. (32)	18	M	3.7	LF	Rejection			
				Re-transplant (6.2 months after 1st LT)	Sepsis Death^h^	0.6		
	19	M	NA	NA	NA	NA	NA	NA
	20	F	NA	NA	NA	NA	NA	NA
Matern et al. (33)	21	M	1.3	LF	None	13.5	NA	None
	22	M	3.7	LF	None	5.5	NA	None
	23	M	5.5	LF	PVT	9.9	NA	None
Troisi et al. (34)	24	F	2	NA	None	0.8	normal	None

LF, liver failure; HA, hepatic adenomas; HF, heart failure; LC, liver cirrhosis; PVT, portal vein thrombosis; LT, liver transplantation; HAT, hepatic artery thrombosis; M, male; F, female; NA, not available.

a Death due to terminal heart failure and Gram-positive bacterial sepsis.

b Death due to heart failure.

c Death 7 days post-LT due to sepsis following bowel perforation.

d Death at 1 month post-LT following hepatic artery thrombosis.

e Death due to meningococcal sepsis.

f Due to non-functioning 1st donor liver.

g Death 0.3 years post LT due to respiratory and circulatory failure secondary to sepsis.

h Death 1 month after the second LT due to sepsis.

On myocardial biopsy, PAS–positive deposits in the myocardium were found in 10 patients with GSD IV, including five postmortem specimens (Table 2). All myocardial biopsies were performed postoperatively, except for biopsy of the first patient. Death was reported in nine (37.5%) patients because of: sepsis following bowel perforation 7 d after transplantation (*n* = 1); meningococcal sepsis (*n* = 1); sepsis 1 mo after second liver transplantation (*n* = 1); respiratory and circulatory failure secondary to sepsis 0.3 years post-transplantation (*n* = 1); terminal heart failure and Gram-positive bacterial sepsis 76 mo after liver transplantation (*n* = 1); cardiomyopathy (*n* = 3); and hepatic artery thrombosis 1 mo post–liver transplantation (*n* = 1). Five patients died <1 year after transplantation;two of whom died <30 d after liver transplantation. Four patients died > 1-year post–transplant. All other patients were alive at time of follow–up (range several months to 13.5 years after transplantation). In all identified survivors, liver function was normal and portal hypertension was decompressed via the new liver. Catch–up growth was reported in eight patients.

**Table 2:** PAS-positive inclusion deposits in the myocardium of ten patients with GSD IV pre-or-post liver transplantation.

**References**	**Case**	**Time sample (months posttransplant)**	**Mean % area occupied by inclusions**	**No of inclusions per 3.6 × 10^**5**^ μm^**2**^**	**Mean size of inclusions (μm^**2**^)**
Aksu et al. (3)	1	Pretransplant	–	–	–
Willot et al. (25)	3	76.0 (Autopsy)	–	–	–
	4	11 (Autopsy)	50%	–	–
Sokal et al. (26)	5	9.0	–	–	–
Selby et al. (23, 28)	7	54.0	0.5%	24	140
	9	1.2 (Autopsy)	2%	46	141
	12	0.9	13 %	332	146
		14.0	5.9%	129	164
	13	1.0		Too few to quantitate	
		11.0		Too few to quantitate	
Alshak et al. (30)Rosenthal et al. (31)	15	30.0 (Autopsy)	–	–	–
Magoulas et al. (2)	17	4.0 (Autopsy)	–	–	–

Cardiomyopathy was the most frequent and serious complication, eventually leading to heart failure. Cardiomyopathy developed in six (25%) patients, including one with pre-transplantation heart failure. None of these individuals had undergone combined liver–heart transplantation. Transplant–related complications were seen in eight (33.3%) patients and included portal vein thrombosis (*n* = 1), hepatic artery thrombosis (*n* = 1), rejection (*n* = 2), sepsis (*n* = 4) and non-function of graft (*n* = 1). One of the eight patients had both rejection and sepsis. Of the 15 (62.5%) survivors, one presented at birth with severe hypotonia and flexion contractures of the hips and articulus. He was reported to have symptomatic improvement with mild hypotonia and arthrogryposis 3 years post–transplantation. Thirteen survivors had no neuromuscular or heart complications during follow-up for as long as 13.5 years. Notably, there was a marked reduction in the amount of amylopectin on myocardial biopsy in one of these patients after transplantation (case 12, Table 1). The patient who underwent liver transplantation for heart failure had not experienced any symptoms of cardiac failure at 6 mo after transplantation (case 1, Table 1).

## Discussion

Our review shows that liver transplantation corrects the primary hepatic enzyme defect, so the graft does not accumulate polyglucosan. Liver transplantation normalized the liver function of all the patients with GSD IV except case 3, thereby improving the survival rate and quality of life of patients. Since then, liver transplantation has improved patients' outcome because metabolic sequelae, such as cardiomyopathy and skeletal myopathy, have not developed post–transplantation in most cases. Most remarkable, eight patients were reported to had retarded growth pre–transplantation, but developed well with normal growth, after liver transplantation (25, 27, 28). As there is no way to compensate for the insufficient enzyme activity, liver transplantation remains the only effective treatment for patients with the progressive hepatic subtype of GSD IV who develop liver failure.

The clinical onset frequently in patients with classical GSD IV is earlier than in atypical individuals (23). We identified that most patients did not develop cardiomyopathy or neuromuscular complications during follow–up of up to 13.5 years (11). Seven of these patients received liver transplantation from living donors, which included five heterozygous and three ABO–incompatible donors, and no mortality or morbidity associated with heterozygosity has yet been observed (3, 24, 27, 32, 34). One of these patents was a 3.7-year-old boy with GSD IV (case 18, Table 1), who underwent first liver transplantation using an ABO–incompatible liver graft from his mother, which led to graft failure due to ABO-incompatibility and was replaced by an ABO–incompatible liver graft from his father 6.2 mo after the first liver transplant. In one patient who underwent liver transplantation for heart failure due to cardiomyopathy, and her heart failure was restored during 6 mo follow–up (3). Remarkably, there was resorption of amylopectin on myocardial biopsy in one patient after liver transplantation (35). The mechanism of this decrease of abnormal glycogen remains unclear (11). It may have been due to migration of donor cells from the liver allograft to the recipient heart and microchimerism (5, 35).

However, the prognosis is grave when extrahepatic manifestations of GSD IV develop, especially cardiomyopathy (36). Accordingly, it is a major concern that liver transplantation may only improve the hepatic function of individuals with GSD IV, and extrahepatic manifestations might develop post–transplantation and result in poor prognosis. Amylopectin is not soluble, and the enzyme defect is present in other affected organs such as muscle, heart or nervous system. Amylopectin accumulation in other affected tissues might progress after transplantation. Postoperative extrahepatic progression of the disease caused by amylopectin accumulation in other affected tissues might be a potential risk for these individuals. Cardiac amylopectionosis was the most common postoperative complication in GSD IV patients (6/24) and four of those died from heart failure. We identified only one patient with heart failure before transplantation. The evaluation of pretransplant cardiac function is not a predicting factor of poor outcome in this situation. Of the four patients who died of cardiac failure after liver transplantation, all preoperative heart function was normal, but they developed cardiac amylopectionosis, attributed to accumulation of amylopectin in cardiac muscle. The patients died 2.55 years (range: 0.9–6.3years) after transplantation (25, 26, 31). In three of those four patients, autopsy showed that cardiomyocytes contained massive PAS–positive, diastase-resistant inclusions (25, 31). In the fourth patient, postoperative myocardial biopsy showed PAS–positive, amylase–resistant deposits in cardiomyocytes up to 9 mo following liver transplantation (26). In another patient, cardiomyopathy was discovered by postmortem examination after he died from respiratory and circulatory failure secondary to sepsis (2). Myocardial biopsy is a potential predictor of cardiac functional prognosis after transplantation, but the number of amylopectin–like deposits related to progressive fatal cardiac failure needs to be defined. Further, long-term follow-up is necessary to evaluate possible cardiac or neuromuscular complications (24). To further evaluate this risk, patients with GSD IV need careful assessment of heart, liver, and muscle before and after liver transplantation.

Heart transplantation has been suggested in cases with severe cardiac involvement. Patients with progressive cardiomyopathy and myocardial involvement confirmed by myocardial biopsy secondary to GSD IV may be candidates for cardiac transplantation. The experience with cardiac transplantation for GSD IV is insufficient. Only three patients are known to have undergone cardiac transplantation for extrahepatic progression related to GSD IV (37–39). Two patients were transplanted successfully and in good condition during follow–up. The third one died due to infectious complications after orthoptic heart transplantation. Evaluation of the genotype–phenotype correlation in GSD IV may be helpful, which may provide valuable information in decision-making and help us to better understand the outcome of liver transplantation (6, 33).

## Conclusion

Liver transplantation remains the only therapeutic option for treatment of hepatic manifestation of GSD IV. Our review shows that all GSD IV patients who survived had normal liver function after liver transplantation. Selection of patients with GSD IV for liver transplantation should be alert to extrahepatic progression, as the cardiomyopathy may lead to fatal complications. Consideration of combined liver–heart transplantation and careful assessment of cardiac function even in the absence of evidence of clinical decompensation appears warranted for patients with GSD IV. Histopathological studies of myocardial tissues and evaluation of the correlation between genotype–phenotype and the condition may predict the degree of severity and assist with treatment decisions.

## Author Contributions

ML identified all the cases to be included, analyzed and interpreted the data and drafted the manuscript. L-YS reviewed the manuscript. Both authors read and approved the final manuscript.

## Conflict of Interest

The authors declare that the research was conducted in the absence of any commercial or financial relationships that could be construed as a potential conflict of interest.
